# Optimization of impedance-measured reflux events in GORD utilizing acid exposure time

**DOI:** 10.1186/s12876-020-01321-z

**Published:** 2020-06-09

**Authors:** Anthony J. Horton, Steven B. Clayton

**Affiliations:** 1 Department of Medicine, Duke University Health System, Durham, NC USA; 2 Gastroenterology and Liver Center, Greenville Health System, Greenville, SC USA; 3 Department of Internal Medicine, Wake Forest University Baptist Medical Center, 1 Medical Center Blvd, Winston-Salem, NC 27157 USA

**Keywords:** Ambulatory reflux monitoring, pH-impedance, Multichannel intraluminal pH, Gastro-oesophageal reflux disease

## Abstract

**Background:**

Combining impedance with pH monitoring improves the detection and characterization of gastro-oesophageal reflux (GOR), yet the two modalities frequently differ in GOR quantification. Ambulatory 24-h pH-impedance monitoring often reveals more significant oesophageal acid exposure than impedance-measured reflux activity in patients with symptomatic gastro-oesophageal reflux disease (GORD). The purpose of this study is to elucidate the discrepancies between these modalities by assessing the predictive accuracy of impedance compared to acid exposure standards.

**Methods:**

A single-institution, retrospective review of sequential 24-h pH-impedance results of 72 patients with symptomatic GOR off anti-secretory therapy was conducted. Reflux events measured by impedance were stratified by patient position and compared to oesophageal acid exposure time (AET). Oesophageal AET limits for GORD detection were utilized as gold standards to generate serial receiver operator characteristics (ROC) curves to assess the sensitivity and specificity of current impedance GORD detection limits and identify optimized impedance standards based on area under the curve (AUC) analysis.

**Results:**

Mean total AET time was 10.5% (± 9.9%), and 63.8% of patients had elevated AET. By impedance, median GOR frequency was 43 (IQR 21–68), and 22.2% exceeded conventional GOR frequency limits of normal. ROC curve analysis revealed the current impedance standard of > 73 GOR events has a sensitivity of 32.6% and specificity of 96.5% (AUC 0.74) for GORD detection. By AUC analysis, an impedance threshold of > 41 GOR events is optimal for GORD detection (sensitivity 69.6%, specificity 80.7%, AUC 0.83).

**Conclusion:**

Conventional impedance standards for abnormal GOR frequency are weakly sensitive for the detection of GORD, providing a possible explanation to discrepancies in AET and impedance interpretation. Lowering impedance-measured GOR frequency limits to > 41 optimizes sensitivity and specificity while increasing congruence between pH and impedance metrics.

## Background

Gastro-oesophageal reflux disease (GORD) is a widely prevalent disorder characterized by the disruption of the esophageal mucosa by, or perceptive sensitivity to, gastric refluxate [1]. GORD is initially diagnosed by clinical suspicion, response to empiric trial of proton pump inhibitor (PPI) therapy, or endoscopic evaluation of the mucosal consequences of refluxate exposure [1]. However, ambulatory reflux monitoring is indicated to provide confirmatory evidence of GORD in patients with atypical GORD symptoms, normal endoscopic evaluation, or prior to considering anti-reflux surgery [1]. Ambulatory 24-h pH-impedance monitoring (24-h pH-imp) is considered the consensus standard for diagnosing GORD, as it provides a quantitative measure of gastro-oesophageal reflux (GOR) events through the detection of oesophageal acid exposure and retrograde movement of liquid or gas refluxate in the oesophagus via impedance monitoring [1, 2].

Among the pH monitoring metrics, oesophageal acid exposure time (AET) is the most reproducible and specific metric to GORD [3, 4]. Elevated AET is not only predictive of a positive response to PPI therapy [5, 6], but multivariate analyses have also shown that elevated AET is the most predictive metric of response to medical and surgical GORD management based on dominant symptom index and global symptom severity score outcomes [7, 8]. Oesophageal multichannel intraluminal impedance (MII) testing provides an adjunct method for assessing GOR by measuring changes in resistance to alternating electrical currents as boluses of liquid, gas, or both pass through the esophagus in retrograde fashion. Impedance is typically used to measure GOR frequency and is correlated with patient symptom perception statistically through symptom association probability (SAP). When combined, 24-h pH-imp allows for the detection of acid, weakly acid, and nonacid reflux events. However, the additional benefits of non-acid reflux detection, which cannot be gleaned from pH studies alone, and SAP correlation is limited to hypersensitive oesophagus phenotypes [9] or when testing on proton pump inhibitor (PPI) therapy [10].

Normal values for AET were initially determined in a study of ambulatory oesophageal pH monitoring that compared patients with the typical GORD symptoms to asymptomatic controls, generating the conventional standards of < 4.2% total AET, < 6.3% AET in the upright position, and < 1.2% AET in the recumbent position [11]. Numerous subsequent studies have found comparable AET cutoffs have a sensitivity of 77–100% and specificity of 85–100% in discriminating esophagitis from normal controls [3, 12–17], and recent consensus guidelines continue to define AET < 4% as definitively normal and AET > 6% as definitively abnormal [1, 18].

Normal values for simultaneous impedance testing were initially determined from serial measurements of impedance measured reflux frequency in 60 healthy volunteers, where the upper 95th percentile was defined as the threshold for the diseased state: > 73 total GOR events, > 67 GER events in the upright position, and > 7 GER events in the recumbent position [19]. However, subsequent studies of reflux frequency have found more heterogeneous results [20], and number of reflux events alone has not been proven to be predictive of treatment outcome [7, 8, 21]. Instead, consensus guidelines recommend GER frequency be used as an adjunctive metric when AET alone is inconclusive [1, 18].

Clinically, we noted frequent discrepancies in 24-h pH-imp performed on patients with symptomatic GOR, often finding a number of cases with abnormal AET and normal impedance-measured reflux events. Given that AET was validated in patients with symptoms of GORD and MII was validated in healthy controls, we sought to analyze the predictive accuracy of impedance findings in relation to AET in patients with GORD symptoms off PPI therapy.

## Methods

### Population

Following approval from the Institutional Review Board of Greenville Health System, a sequential retrospective review of ambulatory 24-h pH-imp studies conducted on patients presenting with typical and atypical symptoms of GORD between May and November of 2016 was performed. Typical symptoms of GORD were defined as heartburn and regurgitation, and atypical symptoms of GORD were defined as chest pain, epigastric pain, cough, throat clearing, dysphagia, globus, belching, and sore throat [22]. Because the aim of the study was to assess impedance metrics in patients with unmedicated, symptomatic GOR, tests performed while on acid suppressive medications were excluded.

### Study conduct and analysis

Ambulatory 24-h reflux testing was performed using a combined pH-MII catheter (MedTronic Inc., Shoreview, MN, USA). Following a fasting period ≥5 h, patients underwent MII esophageal manometry for the localization of the lower oesophageal sphincter (LOS) and assessment of oesophageal function. The combined pH-impedance catheter was subsequently placed in the oesophageal body with a reference pH electrode distal to the LOS and 2 pH monitoring electrodes 5 cm and 20 cm proximal to the LOS. The device also contained 8 impedance rings located at 2, 4, 6, 8, 10, 14, 16, and 18 cm proximal to the LOS. The study catheter was attached to an external ambulatory monitor and worn for 24 h during which the study subjects were encouraged to maintain their normal activities, sleep schedules, and eating habits. In addition to pH and impedance data, the 24-h pH-imp monitor provided information regarding the timing of meals, changes in body position, and timing of GORD symptoms, as noted by the patient. Tests not lasting longer than 21 h were excluded from data analysis.

After the study period, data from each monitor was transferred to a computer and visually analyzed using a commercially available software program (MedTronic Inc., Shoreview, MN, USA). Meals were marked and excluded from reflux analysis. Oesophageal acid exposure was quantified as the percentage of time that the pH detected by the distal pH probe was below 4.0, or AET [11, 23]. Because changes in body position and activity can influence intragastric pressure and thus LOS tone, oesophageal acid exposure was recorded as total AET, AET in the upright position, and AET in the recumbent position. These values were then compared to conventional standards [11]. Impedance-measured reflux episodes were defined as the presence of a retrograde waveform indicating a 50% decline in impedance from baseline at the two most distal impedance sites in the oesophageal body [24, 25]. These reflux episodes were further classified as acid when the distal pH probe noted a concurrent drop in esophageal pH below 4·0, weakly acid when the pH remained between 4.0 and 7.0, or nonacid if the pH remained above 7.0. Impedance reflux events were also stratified by upright or recumbent body position [2, 19]. Each recorded impedance event was manually verified by a gastroenterologist with oesophageal training to limit artifact associated with impedance testing [2, 26, 27].

### Statistical analysis

Data analyses were performed using SAS Enterprise Guide software (SAS Institute, Inc., Cary, NC, USA). Total and positional AET were recorded as mean values with standard deviations listed. As GOR values are discrete numerical entities, impedance data is presented as median values with 25th–75th interquartile ranges (IQR). To assess the predictive accuracy of MII testing, the original pH definitions set by Johnson and Demeester (1974) were used as the gold standards for the detection of pathological levels of reflux events [11]. Thus > 4.2% total AET, > 6.3% AET in the upright position, and > 1.2% AET in the recumbent position were the chosen standards for identifying pathologic levels of GOR, respectively. These values were then used to generate serial receiver operator characteristics (ROC) curves to analyze the conventional impedance cutoffs first described by Shay et al. (2004) and to determine new limits of normal impedance with optimized sensitivity and specificity based on area under the curve (AUC) analyses [19]. In this model, the AUC of each ROC curve was representative of how well patients with abnormal study findings via pH are identified by serial impedance thresholds. Optimal impedance cutoffs for total acid reflux were determined in similar fashion using total AET > 4.2% as the pH standard for analysis.

## Results

### Patient population

In total, 72 patients met inclusion criteria. There was an unequal distribution of men and women, with 75% being female. Patients had a mean age of 55.5 ± 13.2 years (range 26–87), and the average patient was overweight with a BMI of 29.2 ± 5.9 kg/m^2^. The majority of patients presented with at least one typical symptom of GORD; 30% of patients included in this study had isolated atypical symptoms of GORD.

### pH probe findings

**Fig. 1:**
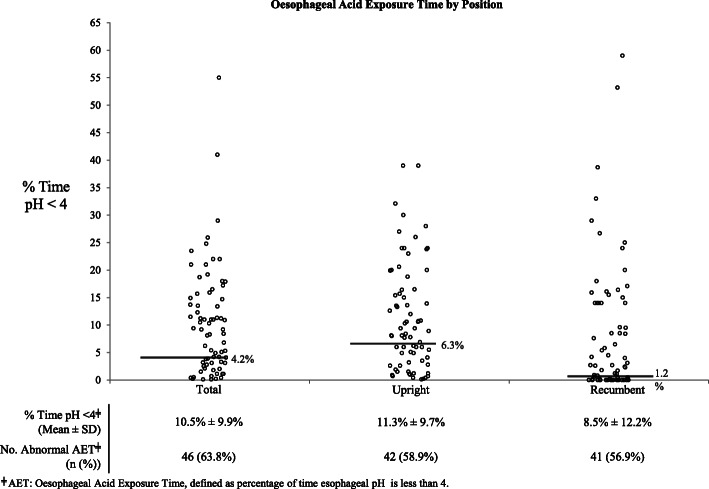
Oesophageal acid exposure time by patient positioning. Horizontal bars are representative of conventional standards for AET [11]

The mean total, upright, and recumbent AET are portrayed in Fig. 1. AET was highest in the upright position (11.3% ± 9.7%), with similar elevations in acid exposure noted in total (10.5% ± 9.9%) and recumbent positions (8.5% ± 12.2%). Individual levels of AET were then compared to conventional thresholds for pathological reflux in scatter plot form (Fig. 1) [11]. Mean total AET was 2.5-fold higher than the conventional threshold of normal, and 63.8% of patients with reflux symptoms had abnormally elevated levels of total esophageal acid exposure.

### Impedance findings

**Fig. 2:**
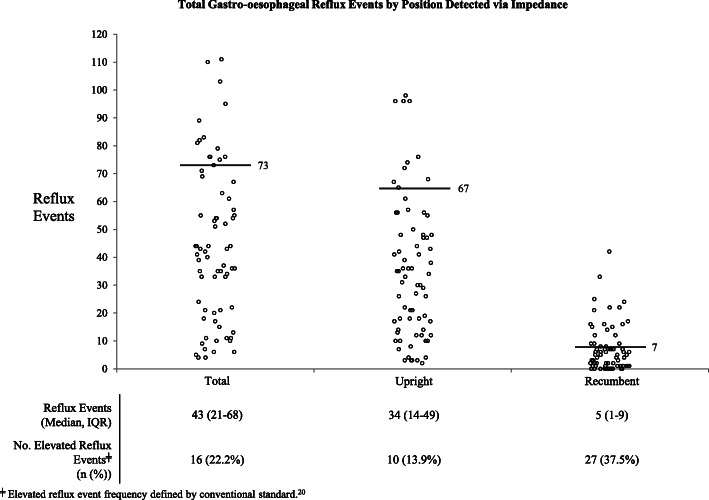
All impedance measured gastro-oesophageal reflux events as stratified by patient position. Horizontal bars are representative of conventional standards for impedance [19]

Median GOR frequency detected via impedance in total, upright, and recumbent positions are portrayed in Fig. 2. Median total GOR frequency was 43 (IQR 21–68), with similar results in the upright position. GOR events in the recumbent position were rare, with a median frequency of 5 (IQR 1–9). Individual reflux frequencies were compared to conventional standards (Fig. 2) [19]. Only 22.2% of patients with GORD symptoms had an abnormally elevated number of total GOR events by these impedance standards.

Simultaneous pH probe measurements allowed for the further characterization of each GOR event (Table 1). Acid GOR occurred at a median frequency of 32 (IQR 13–53), and weakly acid GOR occurred at a median frequency of 7 (IQR 4–14). Nonacid reflux was rare, occurring once in just 3 patients.

**Table 1:**
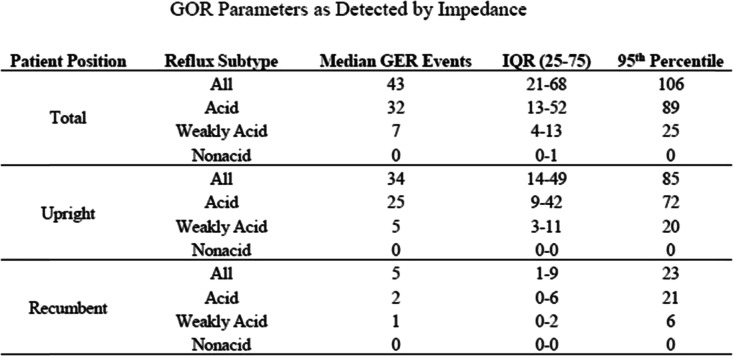
GOR parameters as detected by impedance

Gastro-oesophageal reflux events detected by impedance monitoring in patients with unmedicated, symptomatic GORD. GOR events are stratified by both patient positioning and level of refluxate acidity

### ROC curve analysis

Using AET limits as the gold standard for detecting GOR, the conventional impedance standards were assessed for sensitivity and specificity of disease detection (Table 2). The conventional standard of 73 total impedance measured GOR events had a sensitivity of 32.6% and a specificity of 96.5% for detecting AET levels diagnostic of GORD (AUC 0.74, 95% CI 0.63–0·.86). Similar results are noted for GOR detection in the upright and recumbent positions (Table 2).

**Table 2:**
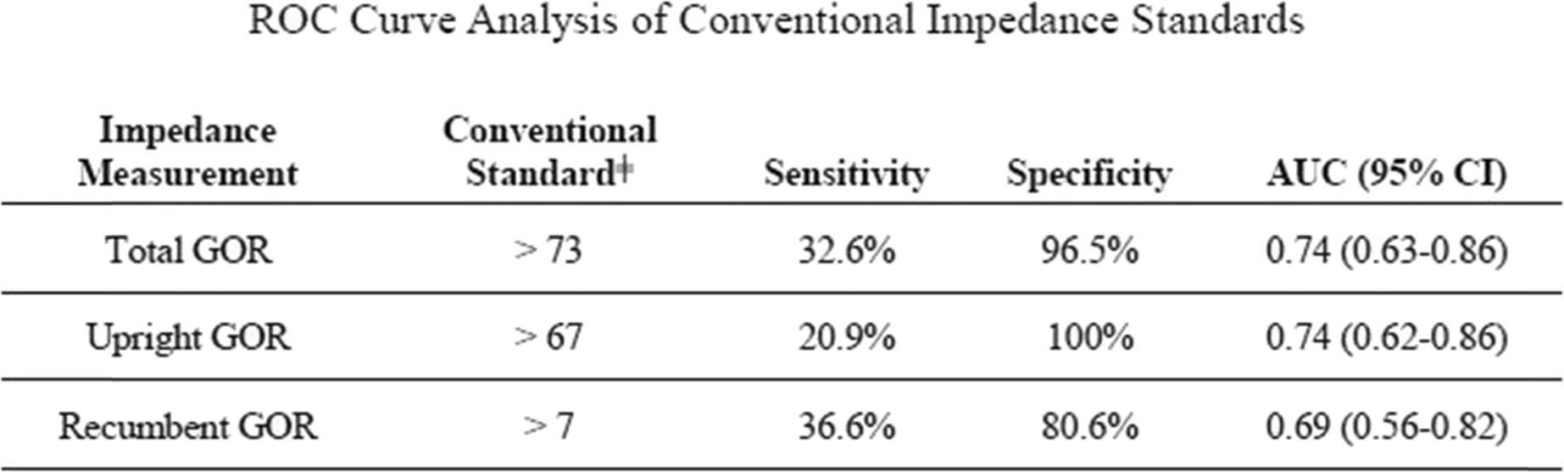
ROC curve analysis of conventional impedance standards

ROC curve analysis of conventional multichannel intraluminal impedance compared to oesophageal acid exposure time standards. Sensitivity and specificity are derived from area under the curve analysis

╪ Elevated reflux event frequency defined by conventional standard [20]

Serial ROC curves were generated to identify impedance cut-offs with optimal sensitivity and specificity as shown in Table 2. By AUC analysis, a threshold of ≥41 total impedance measured GOR events is optimally sensitive and specific for detecting GORD identified by AET (sensitivity 69.6%, specificity 80.7%, AUC 0.83 [95% CI 0.73–0.92]). Optimized impedance thresholds for the upright position and recumbent positions are 29 (AUC 0.84) and 3 (AUC 0.75), respectively.

## Discussion

The present retrospective review of 24-h pH-imp studies provides insight into the clinical discrepancies between the quantifiable severity of pH and impedance findings in unmedicated patients with GORD symptoms. In this sample, average total AET was 10.5 ± 9.9%, which is 2.5-fold higher than consensus AET criteria, and 63.8% of patients met pH criteria for a diagnosis of GORD (Fig. 1). However, only 22.2% of patients met MII criteria for pathologic reflux frequency (Fig. 2) despite a high median total GOR frequency of 43 (IQR 21–68) compared to validated MII studies in asymptomatic controls (Table 3) [19, 28].

**Table 3:**
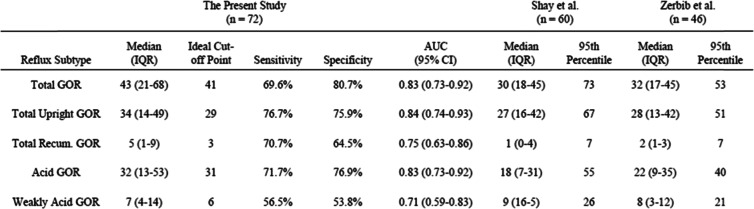
Comparison of impedance findings between the present study in patients with gastro-oesophageal reflux symptoms and previous analyses in asymptomatic patients. The ideal cut-off points for impedance as measured by the present study fall below the conventional cut-offs as determined by the 95th percentile of asymptomatic patients [12, 20]

A review of the historical validation of ambulatory pH and MII testing demonstrates interesting differences between the modalities. Normal values for AET were initially validated through direct comparisons between patients with and without GORD symptoms [11], and subsequent studies found comparable AET cutoffs with reliable sensitivity and specificity for GORD [3, 12–17]. Using AET as a primary metric, additional studies of pH alone or combined pH and MII monitoring have indicated that elevations in AET is the most predictive marker of response to PPI therapy [5, 6] improvement in symptom burden [7], and post-surgical outcomes [8]. On the other hand, MII was initially validated in asymptomatic volunteers, on and off PPI therapy, and stratified based on 95th percentiles to define pathologic GOR frequency [19]. Furthermore, subsequent impedance studies have been more widely heterogeneous without a clear cut-off for abnormal GOR frequency [10, 28–30], and GOR event frequency has not been clearly shown to affect GORD treatment outcomes [7, 8, 21].

With this in mind, we generated a novel analysis of the predictive accuracy of GOR frequency to identify AET-defined GORD using ROC curves (Table 2). In this model, current thresholds for abnormal MII GOR frequency are weakly sensitive (20.9–36.6%) and highly specific (80.6–100%) (Table 2). This weak sensitivity for reflux is congruent with the sub-diagnostic levels of impedance noted in our panel of patients with symptomatic GOR and may offer insight into the poor clinical outcomes data assessing GOR frequency changes in the diseased state. Additionally, the AUC for current MII GOR thresholds is low, suggesting impedance GOR measures tend to underdiagnose GORD and thus may be less useful as an adjunctive metric per the Lyon Consensus guidelines [18].

Using the same model, AUC analyses of serial ROC curves identified a MII GOR frequency cut-off of ≥41 events has an optimized AUC of 0·83 with a sensitivity of 69.6% and specificity 80.7% for detecting pathologic levels of GOR by pH analysis (Table 3). A cut-off of 41 reflux events falls significantly below current consensus thresholds [1, 18], but is higher than the median frequency of events seen in validated studies of healthy controls (Table 3) [19, 28]. In this sample, lowering the GOR frequency threshold from ≥73 events to ≥41 events would increase the percentage of patients meeting GORD criteria from 22.2 to 52.7%, a number more closely approximating the incidence of elevated AET (63.8%) in the same population. A threshold of < 41 events also aligns more closely with the Lyon Consensus definition of < 40 reflux events as definitively physiologic [18]. Reducing the threshold from ≥73 events to ≥41 events to would also raise the Cohen Kappa coefficient (κ) of inter-test reliability between AET and GOR frequency from 0.25 to 0.38. In practice, expanding the correlation between impedance and pH will help alleviate the clinical conundrum that arises when the independent assessment of pH and MII metrics differs in interpretation, thereby increasing the diagnostic confidence of the clinician and leading to more disease directed therapy.

Limiting analysis of the accuracy of MII GOR detection is a lack of a true gold standard for diagnosing GORD given its broad spectrum of diseased state ranges from functional heartburn to Barrett’s oesophagus [18, 31]. In lieu of this, impedance was compared directly to AET due to the aforementioned reproducibility, reliability, predictive value, and prospective significance of the metric. However, a not insignificant portion of patients with typical and atypical symptoms of GORD as a result of non-erosive reflux disease, oesophageal hypersensitivity to refluxate exposure, and functional heartburn may be missed by AET detection alone, and the benefits of quantifying and characterizing non-acid GOR events have been described [9]. Still, it should be noted that in 24-h pH-imp studies conducted while off acid suppressive therapy for a period of ≥7 days, the majority of reflux events are acidic [10, 32–35]. This finding was echoed in the present study in which nonacid reflux occurred in 3 patients at a frequency of 1 event per individual. Therefore, in a population of patients with symptomatic unmedicated GORD, the major benefit of MII to detect nonacid GOR may be negligible; thus, the use of pH-based definitions of pathological reflux may be viewed as an acceptable standard [2, 19, 36, 37].

The Lyon Consensus also summarizes novel metrics that can be obtained from ambulatory pH-impedance testing, including the post-reflux swallow-induced peristaltic wave (PSPW) index and mean nocturnal baseline impedance (MNBI) [18]. While the PSPW index has shown promise in augmenting the diagnostic value of ambulatory pH-impedance testing, it requires an additional cumbersome manual calculation of the ratio of impedance measured reflux events that are followed by a PSPW wave. This value has been shown to correlate with esophageal body peristaltic reserve as well as discriminate pathologic acid exposure form non-pathologic acid exposure states (reflux hypersensitivity, functional heartburn, and control patients) [38–40]. The aim and design of this study was to compare total impedance measured reflux event numbers generated by ambulatory reflux monitoring software (with manual verification) in high and low acid exposure states, and interpretation of the PSPW index would not be affected by changing the thresholds of normality for GOR events. Likewise, MNBI has also shown promise as a surrogate measure of the microscopic changes caused by oesophageal acid exposure that can predict relative AET and response to antireflux therapies without impact from changing GOR frequency thresholds [39, 41–43]. As evaluating these novel metrics was not the focus of the present study, these parameters were not examined.

Lowering MII GOR frequency thresholds will inevitably increase the number of false positive GORD diagnoses. While this may be an acceptable risk to increase the concordance between pH and impedance metrics and a more simplified analytical process, one may also opt to utilize baseline impedance or post-reflux swallow induced peristaltic wave analysis when a diagnosis of GORD is still in question [1, 18]. Moving forward, a prospective study utilizing these proposed cut-off values in symptomatic patients will help to establish a true sensitivity and specificity for these values.

## Conclusions

Patients with GORD symptoms who undergo 24-h pH-imp testing off of acid-suppressive therapy are expected to have elevated levels of total oesophageal AET in a true diseased state, but they frequently will not have a GOR frequency that meets diagnostic criteria by current MII standards. Conflicting pH and impedance results can create a diagnostic dilemma that may delay or alter appropriate treatment for GORD. Using ROC curve analysis with AET as the standard for GORD identification, we have concluded that the current conventional standards for MII GOR frequency are weakly sensitive. This suggests current impedance normative values underdiagnose GORD and may be partially responsible for the discrepancy in pH and impedance results seen clinically. By lowering GOR frequency thresholds, MII may be optimized to detect GORD in symptomatic patients. Future prospective studies with these new thresholds should be performed to validate these retrospective findings.

## Data Availability

All de-identified data and materials pertaining to this study are available for review from the corresponding author on reasonable request.

## Abbreviations

24-h pH-imp: 24-hour pH-impedance monitoring
AUC: Area under the curve
AET: Acid exposure time
GOR: Gastroesophageal reflux
GORD: Gastroesophageal reflux disease
IQR: Interquartile range
LOS: Lower esophageal sphincter
MII: Multichannel intraluminal impedance
PPI: Proton pump inhibitor
ROC: Receiver operator characteristics
SAP: Symptom association probability
